# Thermal Effect on the Formation of Polyethylene Greenhouse Plastiglomerates from Microplastics

**DOI:** 10.1021/acsomega.6c05760

**Published:** 2026-08-03

**Authors:** Luísa Carvalho Pereira Araújo, Fábio Minoru Yamaji, Franciane Pádua

**Affiliations:** Department of Environmental Sciences, Federal University of São Carlos (UFSCar), Campus of Sorocaba, Rodovia João Leme dos Santos, km 110, Sorocaba, São Paulo 18052-780, Brazil

## Abstract

This study examined the impact of photodegradation and thermal degradation on polyethylene (PE) microplastics (MPs) through the analysis of their wettability, surface morphology, sand adhesion, and physical changes after exposure to UV radiation (72 and 130 h) followed by heating at 250 °C. The results of water contact angle (WCA) measurements indicated that photodegradation reduced hydrophobicity, with the sample photodegraded for 130 h (P130) exhibiting the lowest angle (64.4°) prior to heating. After heating, all samples showed a decrease in hydrophobicity, except for the P130 sample, which exhibited a slight increase in the contact angle. Scanning electron microscopy (SEM) analysis revealed changes in the polymer structure that became more pronounced with increasing photodegradation time, with the P130 sample showing more significant surface damage. The formation of plastiglomerates was observed, particularly in the samples photodegraded for 72 h (P72), as these samples exhibited greater adhesion to sand due to the presence of more polar functional groups, such as carbonyls. The P72 sample showed the greatest weight increase (1659%) after sand adhesion, while the original sample exhibited the largest area reduction (72%) after heating. FTIR analysis confirmed the formation of oxidized groups, such as carbonyls and hydroxyls, in the photodegraded samples and highlighted the effects of photodegradation and thermal degradation on the polymer structure. This research demonstrates the combined influence of photodegradation and thermal stress on the physical and chemical properties of PE microplastics.

## Graphical abstract

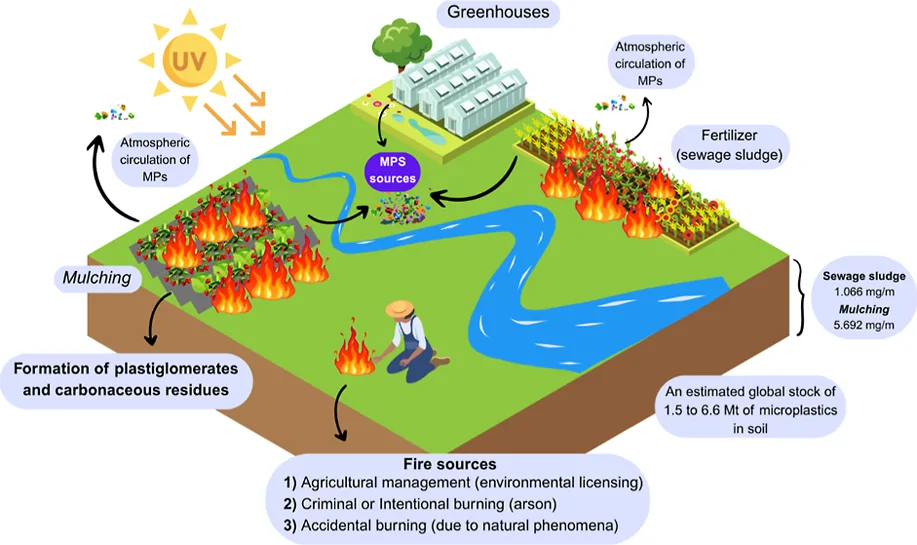

## 1 Introduction

In 2024, Brazil experienced a wave of wildfires that devastated the country, serving as one example of the complex environmental crises plaguing the planet. In August 2024 alone, more than 5.5 million hectares were heated.^1–3^ The combination of rising temperatures, prolonged droughts, and environmental degradation, intensified by human activities, has led to a significant increase in the frequency and severity of wildfires, particularly in Brazilian biomes, namely the Amazon, Pantanal, and Cerrado.^4^ A study demonstrated that climate change caused by human activities increased the intensity of fires in the Pantanal by 40% in 2024.^5^ These Brazilian biomes, known for their biodiversity and crucial role in the national climate balance, have been increasingly affected by climate change6 and global warming.^5–7^

As a result, global warming, driven by greenhouse gas emissions, has accelerated climate change, leading to extreme weather events such as heat waves and severe droughts. In Brazil, these conditions create an environment conducive to forest fires, for example, which have become increasingly difficult to control. In addition, deforestation and improper land use, especially the practice of heating to expand agricultural land, also exacerbate the situation, destroying large areas of forest and releasing massive amounts of carbon dioxide into the atmosphere, further contributing to global warming.^7^

This scenario of wildfires not only destroys vegetation and living organisms, but also impacts soil quality and its ability to regenerate. One of the least visible yet most concerning side effects is the formation of plastiglomerates, a mixture of melted plastic and sediments, such as sand and organic debris, resulting from high temperatures. During fires, extreme heat can melt plastic waste present in the soil, especially in more urbanized areas or those near human activities, creating plastic aggregates that become integrated into the natural environment. The first detection was at Kamilo Beach in Hawaii,^8^ and based on this discovery, the term “plastiglomerate” was coined for this new form of plastic pollution. In Brazil, the first plastiglomerate was found on Trindade Island, in the state of Espírito Santo.^9^ Since then, other plastiglomerates have been detected, mainly in natural environments, particularly in coastal areas.^10–12^

However, most of the plastiglomerates studied range in size from meters to millimeters, without taking into account the micron scale, which encompasses microplastics (MPs) and is one of the major current concerns in the field of plastic pollution.^13,14^ Several recent studies have already indicated that MPs present in the soil can impact soil structure and fertility, interfere with nutrient uptake by plants, and affect organisms essential to ecosystem health.^13,15–22^ Furthermore, the primary source of MPs in soils stems from agricultural plastics, such as greenhouse polyethylene, and sewage sludge fertilizer. Given the correlation between the presence of MP in soil, with a global stock estimated at 1.5 to 6.6 megatons,^16,19^ and increasingly frequent wildfires, research aimed at understanding the impacts of these new plastic formations has become essential. Therefore, the objective of this study is to investigate the effect of fire on the formation of plastiglomerates from greenhouse polyethylene microplastics.

## 2 Materials and Methods

### 2.1 Polymer

The polymer used as the raw material was greenhouse-grade polyethylene in its pristine state. The sand used as the surface for thermal treatment the polyethylene was dolomitic sand, commonly used in aquariums. For each thermal test, 200 g of sand was used as the standard.

### 2.2 Production of MPs

The microplastics were manually cut into pieces approximately 5 mm × 5 mm in size. The larger size of MPs^23^ was chosen so that their behavior before and after the thermal test could be observed macroscopically, i.e., with the naked eye. All samples were prepared in duplicate, and their area and mass were measured before and after thermal treatment, as shown in Tables 1 and 2.

**Table 1:** Results of the Two-Way ANOVA for the Effects of Photodegradation Stage and Heating Condition on the WCA MPs Samples

source of variation	d*F*	*F*	*p*-value
photodegradation (stage)	2	8983.35	<0.0001
heating (condition)	1	2057.35	<0.0001
stage × condition interaction	2	764.93	<0.0001
residual (error)	162	—	—

**Table 2:** Tukey’s HSD Post Hoc Test Comparing Heated and Unheated Polyethylene Samples within Each Photodegradation Stage^a^

comparison	mean difference (°)	adjusted *p*-value	significance
P0 × P0 heated	7.63	<0.001	***
P72 × P72 heated	6.46	<0.001	***
P130 × P130 heated	0.92	<0.001	***

a Significance levels are indicated as follows: ns, not significant (*p* ≥ 0.05); *p < 0.05; **p < 0.01; ***p < 0.001; ****p < 0.0001.

### 2.3 Photodegradation Chamber

Pristine polyethylene microplastics (MPs) were exposed to a UV light chamber containing three 36 W Osram HNLS L lamps for 72 and 130 h.^24^

The samples were designated P0, P72, and P130, based on their respective photodegradation times: P0 (0 h), P72 (72 h), and P130 (130 h).

### 2.4 Thermal Treatment

A Jung brand muffle furnace was used and set to a temperature of 250 °C. This temperature was chosen because many studies^25,26^ consider three temperature ranges based on soil depth: 250 °C, 350 °C, and 500 °C, which correspond to low, medium, and high temperatures, respectively. Therefore, in this study, we began with the low temperature.^26–28^

After that, 200 g of sand were placed in a metal container for each thermal test, and the samples of virgin MP, as well as those photodegraded for 72 and 130 h, were placed on top of the sand. The samples were then placed in an oven at a temperature of 250 °C for 30 min^27^ After heating, the samples were removed from the oven and allowed to reach room temperature to begin measurements and characterization.

### 2.5 Measurement of Sample Mass and Area

All samples were weighed before and after thermal treatment, and the average weight is presented in Table 1. The area was measured using ImageJ software, with the same standard threshold of 172 × 255 for all samples measured. Furthermore, statistical analysis was performed using one-way ANOVA, followed by Tukey’s post hoc test to identify significant differences between groups.

### 2.6 Fourier-Transform Infrared Spectroscopy (FTIR)

FTIR analysis was performed at the UFSCar Lignocellulosic Materials Laboratory in Sorocaba. The microplastics (MPs), before and after thermal treatment, were inserted into the instrument’s sample holder and analyzed in the wavenumber range of 4000 to 400 cm^–1^, using an attenuated total reflectance (ATR) accessory, with a resolution of 4 cm^–1^ and 32 scans. The instrument used was the Agilent Cary 630 FTIR.

The spectra were normalized using OriginPro 9 software, with Min-Max normalization,^28,29^ according to eq 1. The spectral values were scaled to the range [0, 1]. According to eq 1, the values *y*_min_ and *y*_max_ represent the minimum and maximum values of the data set, respectively.

1 $$y^{\prime }=\frac{y-y_{\min}}{y_{\max}-y_{\min}}$$

### 2.7 Water Contact Angle (WCA)

The analysis was performed using a Ramé-Hart 100–25 M goniometer at the Laboratory of Electrochemical Optical Characterization at UFSCar Sorocaba. The samples were heated on a metal plate at 250 °C in a muffle furnace with virgin microparticles (MPs), photodegraded for 72 and 130 h. After this period, the surface wettability was determined by measuring the static contact angles in triplicate, resulting in 30 measurements for each sample. For this evaluation, 5 μL water droplets were deposited on the surface of the MPs, and images were captured. To verify the significance of the results, a one-way ANOVA test followed by Tukey’s post hoc test was performed.

### 2.8 Scanning Electron Microscopy (SEM)

Conducted at the National Nanotechnology Laboratory (LNNano), located at the National Center for Research in Energy and Materials (CNPEM) in Campinas. Backscattered electron (BSE) and secondary electron (SE) modes were used.

## 3 Results and Discussion

### 3.1 Fourier Transform Infrared Spectroscopy (FTIR)

Through FTIR analysis, as shown in Figure 1, it is possible to identify the main bands related to the photodegradation of PE. In the wavelength range of 1710–1740 cm^–1^, carbonyl groups (C=O stretching) can be observed. The appearance of this band is generally due to the formation of carbonyl-containing degradation products, such as ketones, aldehydes, and carboxylic acids.^30^ It is a primary indicator of oxidative degradation.^31^ Similarly, at the wavelength range of 1170–1250 cm^–1^, ester groups (C–O stretching) can be observed. These bands may arise due to the formation of ester-like structures during degradation, also indicating oxidative changes in the polymer.^32,33^

**Figure 1:**
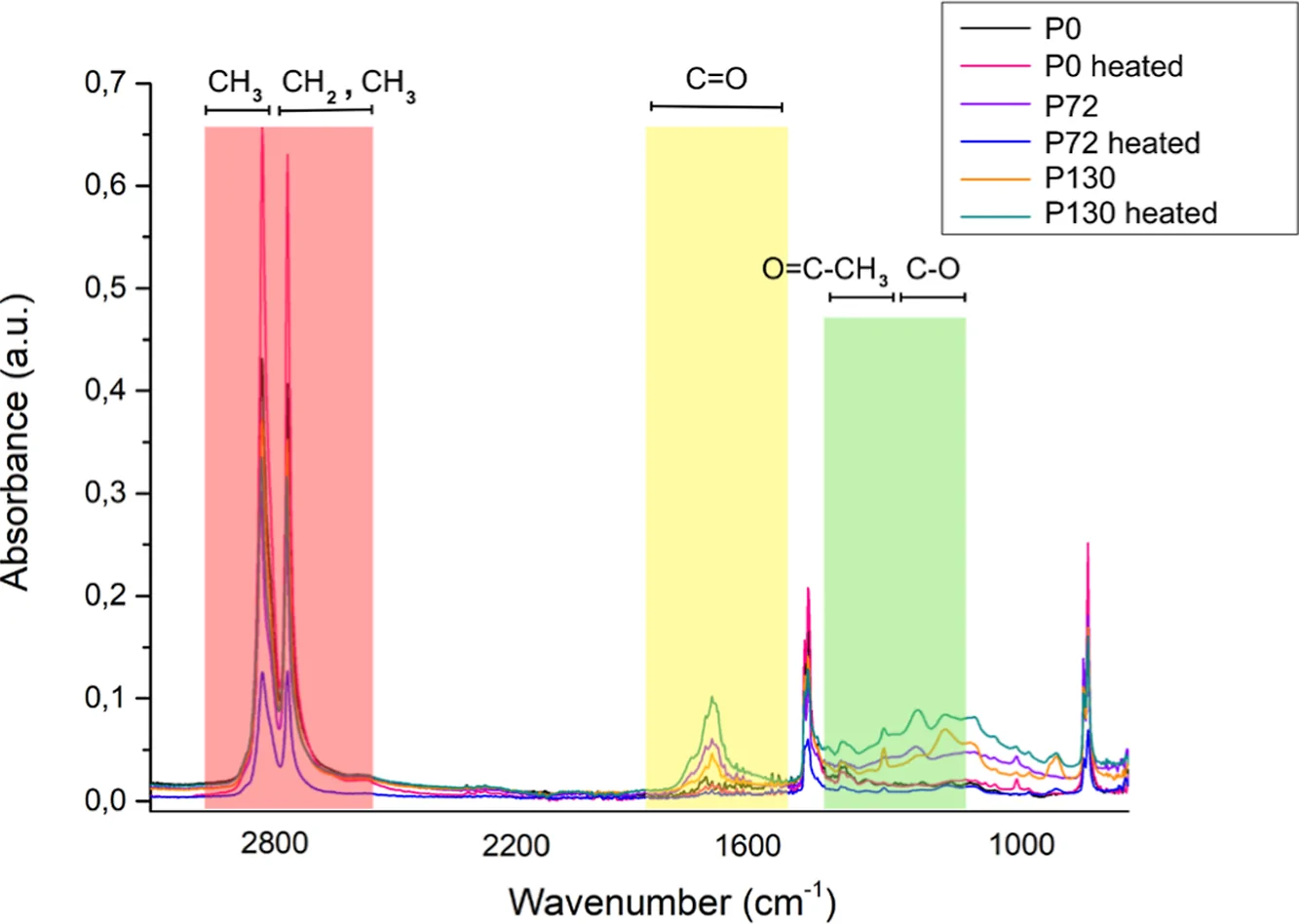
FTIR spectra of all MP samples, before and after heating at 250 °C.

In addition, as shown in Figure 2, there are also methylene groups (CH_2_) present at 2850–2920 cm^–1^ (asymmetric stretching) and 1460–1470 cm^–1^ (bending), which are significant because changes in the intensity of these bands may indicate chain scission or cross-linking in the polymer structure during photodegradation. Among other organic groups, there are also hydroperoxides (O–O stretching), with a wavenumber of 860–880 cm^–1^, which are transient products of the photodegradation process, generally observed in the early stages of oxidation.^34^

**Figure 2:**
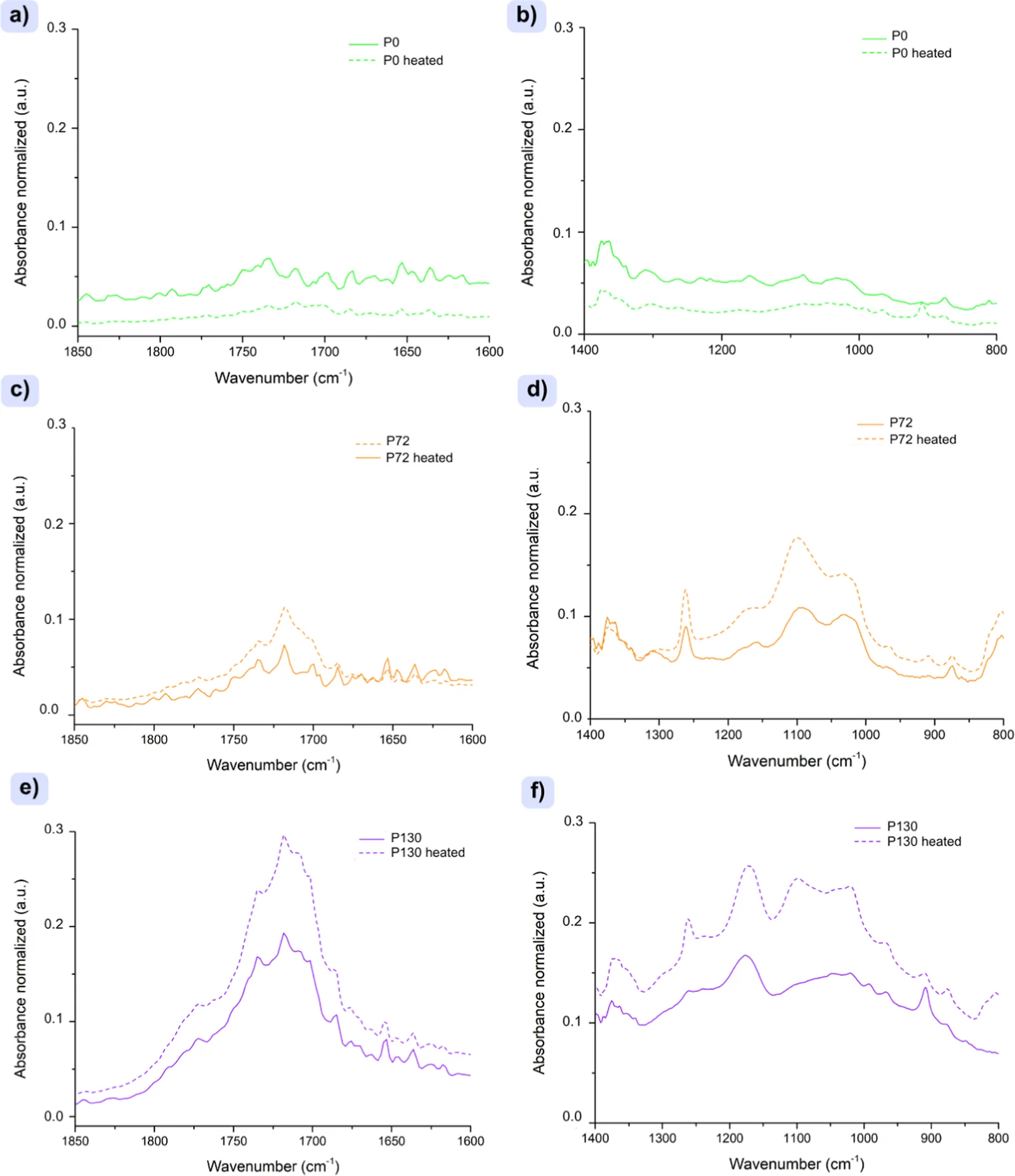
FTIR spectra of the MP samples before and after thermal treatment at 250 °C. Highlighting the main bands related to PE degradation, carbonyl groups, and ester groups. In Figure 2 (a,c,e), the carbonyl groups in the samples are indicated, while in 2 (b,d,f), the ester groups are shown.

As shown in Figure 2, qualitative differences can be observed in the carbonyl region among the samples. Although the spectra suggest a tendency toward higher carbonyl absorption after photodegradation and subsequent heating. Therefore, the FTIR results should be interpreted as qualitative evidence of oxidation rather than definitive proof of increasing degradation. In this way, in Figure 2, the carbonyl region exhibits a progressive increase in absorbance with increasing exposure time to photodegradation, indicating the continuous formation of oxygenated species, such as ketones, aldehydes, carboxylic acids, and ester structures. This behavior is consistent with what is expected for polyolefins subjected to environmental aging, in which photoinduced oxidation is responsible for the breakdown of polymer chains and the subsequent incorporation of oxygen.^35^

From a comparative perspective, the three samples evaluated P0, P72, and P130 exhibit similar behavior. P0 exhibits a spectrum, Figure 2a, that is practically free of carbonyl bands, reflecting its chemically stable nature prior to exposure to degradative agents. In contrast, sample P72 already reveals an enrichment of oxidative groups, evidenced by the increased carbonyl band in Figure 2c, which characterizes the onset of surface oxidation. Among the analyzed samples, P130 stands out as the most degraded, Figure 2e,f, exhibiting the highest absorbance intensity in the carbonyl region, consistent with the most advanced stage of photodegradation.^36^

The effect of thermal stress at 250 °C acts as an additional factor in the material’s oxidative evolution. The application of heat promotes structural rearrangements, the volatilization of low-molecular-weight chains, and secondary oxidation, resulting in an even more pronounced increase in the carbonyl and ester bands in the previously photodegraded samples. Figure 2e,f shows that P130, subsequently subjected to thermal stress, exhibits the highest carbonyl peak among all tested conditions, indicating that the combination of prior photooxidation and heating significantly intensifies the oxidative process.^37^

The behavior of P0 in Figure 2a,b upon heating, on the other hand, does not show such pronounced bands. Although there is some increase in the carbonyl region, it is considerably lower than that observed for the prephotodegraded samples, indicating that the absence of initial reactive sites limits the extent of thermal oxidation. On the other hand, P72 exhibits intermediate behavior; after thermal stress, its carbonyl and ester bands increase noticeably, though still to a lesser extent than observed in the P130 material. This progression demonstrates that the extent of thermal oxidation depends directly on the degree of prior photodegradation.^38^

The ascending qualitative order of intensity of the carboxyl region shown in Figure 2 can be described as follows: P0 (heated) < P0 (unheated) < P72 (unheated) < P72 (heated) < P130 (unheated) < P130 (heated). This sequence highlights the relationship between the effects of photodegradation and thermal stress, in which the prior presence of free radicals, surface cracks, and partially oxidized structures facilitates secondary oxidation during heating.^39^

In summary, the results presented confirm that both photodegradation and thermal stress act as key agents in the generation and amplification of carbonyl groups and esters. Photodegradation progressively increases the susceptibility of PE to oxidation, while thermal stress intensifies this effect, especially in samples that are already degraded. Thus, the comparative analysis of the samples reinforces that the greatest oxidative progression occurs when both processes are combined, resulting in more profound chemical transformations and an intensification of the characteristic bands observed in Figure 2.^40,41^

### 3.2 Contact Angle with Water (WCA)

According to Figures 3 and 4, which shows the samples before thermal treatment, Figure 3a exhibited the largest contact angle (83.1° ± 0.13), indicating a more hydrophobic surface compared to the photodegraded samples, Figure 3c,e. Photodegradation, which occurs due to exposure to UV rays, causes oxidation of the polyethylene surface, forming polar groups such as carbonyls, which increase the surface’s affinity for water, thereby reducing the contact angle. Thus, the contact angle gradually decreases with increasing photodegradation time, with sample P130, Figure 3e, exhibiting the smallest angle (64.4° ± 1.53).

**Figure 3:**
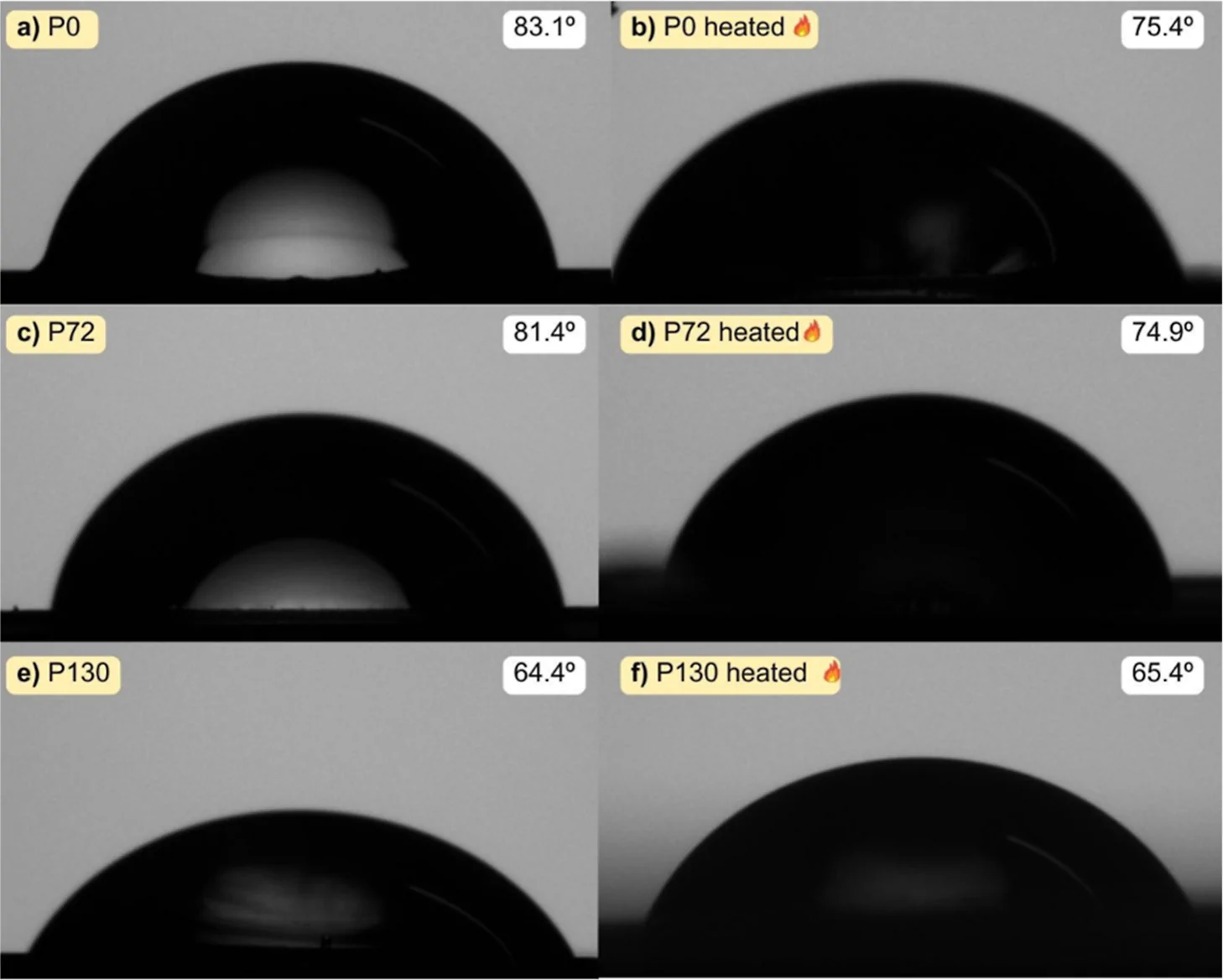
Water contact angle of the samples before and after heating at 250 °C. Where (a,c,e) are the pristine samples, photodegraded for 72 and 130 h, respectively, before heating, and (b,d,f) are the original samples, photodegraded for 72 and 130 h, respectively, after heating.

**Figure 4:**
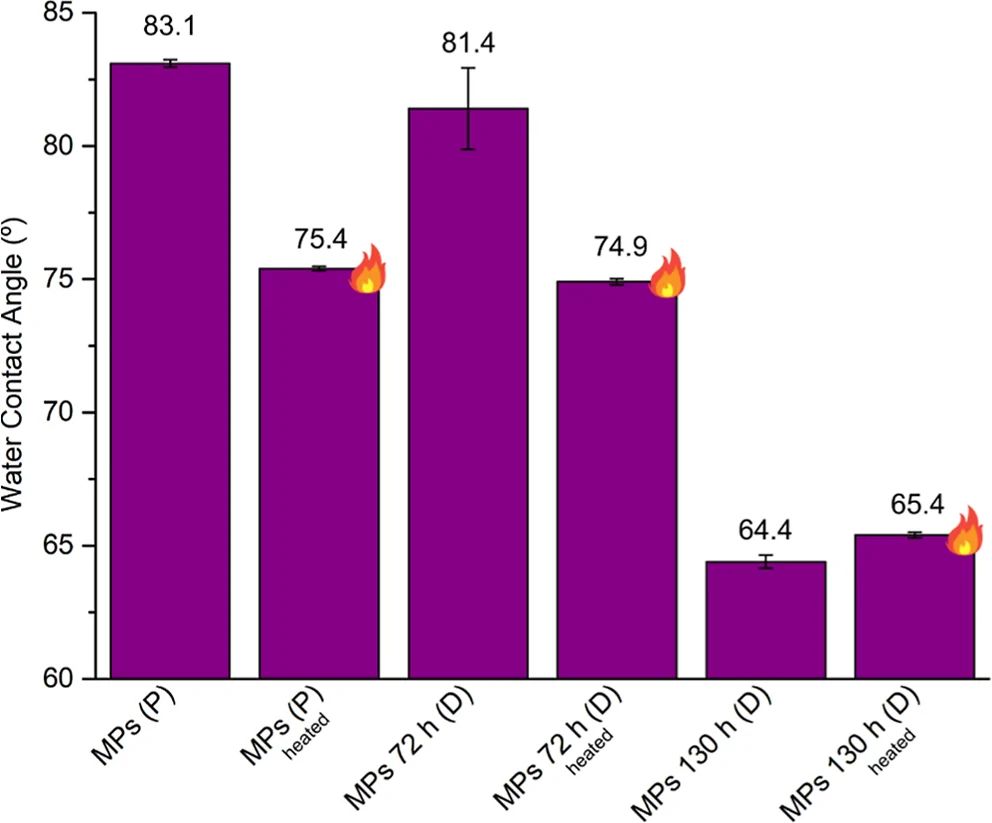
Comparison of the water contact angle (WCA) of the samples before and after thermal treatment at 250 °C. The 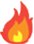 symbol represents the samples after thermal treatment.

According to Figure 3b,d, the samples had slightly smaller contact angles for P0 (75.4° ± 0.12) and for P72, both after heating, which can be explained by the removal of oxidized features, but with the emergence of a rougher and more heterogeneous surface, which maintains hydrophobicity at a moderate level. In the case of P130, Figure 3f, the contact angle decreased (65.4° ± 0.24), indicating that thermal treatment did not completely reverse the effects of more intense photodegradation, but caused some modification in the surface structure.

In summary, the graph in Figure 4 shows that the contact angles after thermal treatment are smaller compared to the values before thermal treatment, suggesting that thermal degradation slightly reduced the hydrophobicity of the samples. The only exception was the heated sample P130, whose angle (65.4° ± 0.24) was greater than that of P130 (64.4° ± 1.53) before thermal treatment, but with only a small variation of 1°, indicating little change in the sample’s hydrophobicity after thermal treatment.^42^

Although sample P130 exhibited an FTIR spectrum with more intense bands in the carbonyl region after heating, indicating greater oxidation. This suggests that the material had already reached a high level of hydrophilicity prior to heating, possibly due to surface saturation with polar groups generated by prolonged photodegradation. Recent studies indicate that, at certain stages of polyethylene oxidation, the formation of hydrophilic functional groups tends to stabilize, limiting further changes in the contact angle with water, even in the face of new thermal processes.^43^

According Table 1, Two-way ANOVA demonstrated that both photodegradation stage, *F*(2,162) = 8983.35, *p* < 0.0001, and heating condition, *F*(1,162) = 2057.35, *p* < 0.0001, significantly affected the WCA. In addition, a highly significant interaction between photodegradation stage and heating condition was detected, *F*(2,162) = 764.93, *p* < 0.0001, indicating that the impact of heating on the surface wettability of polyethylene varied according to the extent of photodegradation.

Following the two-way ANOVA, Tukey’s HSD post hoc test was performed to compare heated and unheated MPs samples within each photodegradation stage, as sown in Table 2. Heating significantly decreased the WCA of P0 (mean difference = 7.63°, *p* < 0.001) and P72 photodegraded polyethylene (mean difference = 6.46°, *p* < 0.001). Although a statistically significant difference was also observed for the 130 h photodegraded polyethylene (mean difference = 0.92°, *p* < 0.001), the effect size was substantially smaller, suggesting that prolonged photodegradation had already altered the surface properties of the material, thereby limiting the additional impact of heating.

### 3.3 Plastiglomerates

#### 3.3.1 Scanning Electron Microscopy (SEM)

As suggests in Figure 5, the formation of some roughness in the PE, visible in scanning electron microscopy (SEM) analyses, reflects the weakening of the material, which becomes stiffer and more brittle due to the decrease in molecular weight and the increase in the concentration of defects in the polymer network. This increase can be observed in ascending order in Figure 5, starting with the pristine sample 5 (a), followed by the sample 5 (b) photodegraded for 72 h, and finally the sample 5 (c) photodegraded for 130 h, respectively. In the photodegraded MPs, these cracks are more pronounced due to greater oxidation and the loss of internal cohesion of the polymer matrix.

**Figure 5:**
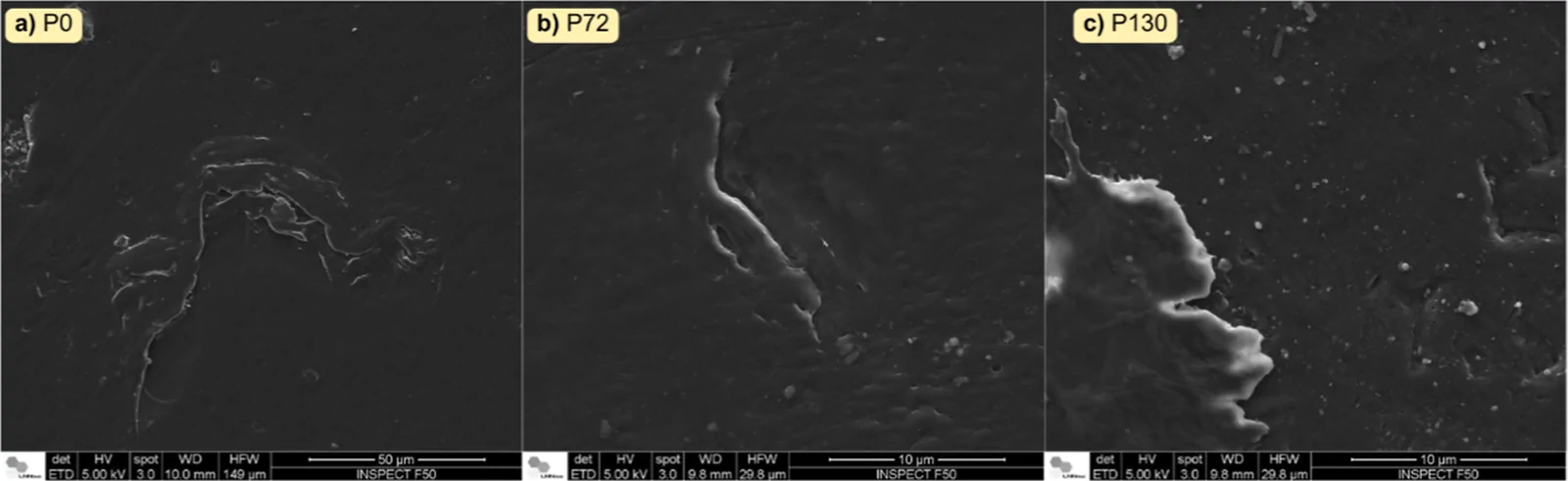
Samples of MPs analyzed by SEM, where (a) 2000× magnification, (b) 10,000× magnification, and (c) 10,000× magnification show the original samples, which were photodegraded for 72 and 130 h, respectively, prior to thermal treatment.

Furthermore, when PE MPs are exposed to UV radiation, the energy is sufficient to break the C–H and C–C bonds in the polymer structure, forming free radicals. These radicals react with oxygen, forming groups such as carbonyl and hydroxyl groups on the polymer surface. Over time, this oxidative degradation weakens the material’s integrity, making it more susceptible to wear.^44^

The adhesion of sand and the formation of plastiglomerates, as shown in Figure 6, in the MP samples after heating to 250 °C, can be explained by the physical transformations of the material during exposure to heat.

**Figure 6:**
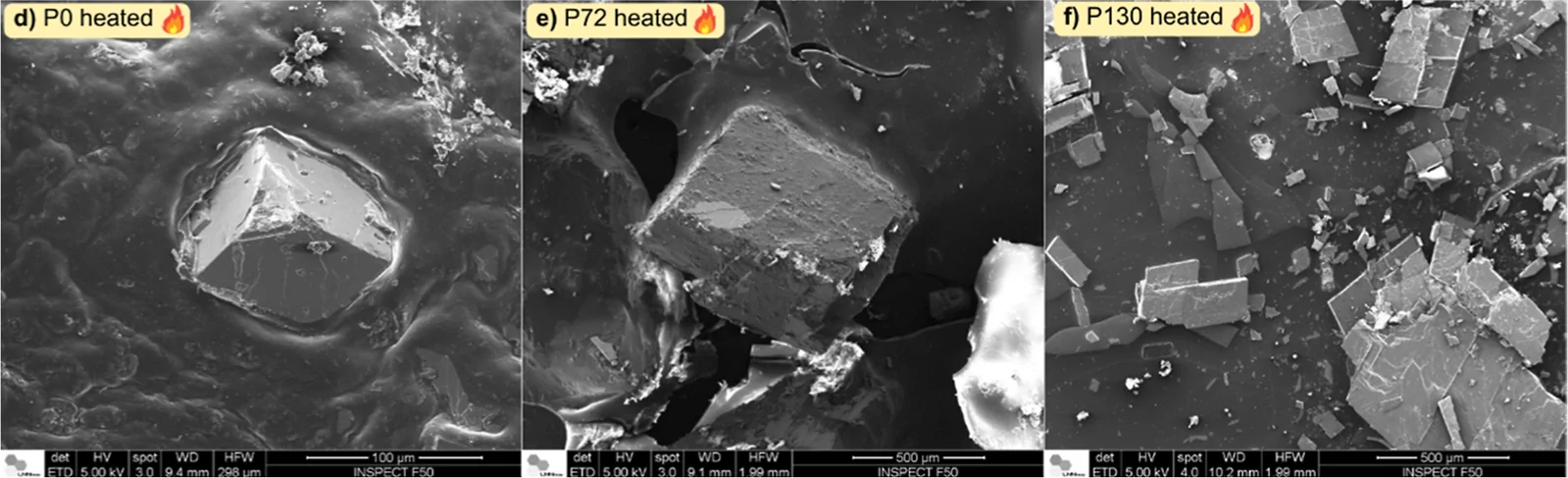
Samples of heated MPs analyzed by SEM, where (d) 1000× magnification, (e) 150× magnification, and (f) 150× magnification show the intact samples, those photodegraded for 72 h, and those photodegraded for 130 h, respectively, following thermal treatment.

When heated, PE undergoes a softening process in which the polymer chains, begin to reorganize and melt, forming agglomerates called plastiglomerates. During this phase, the material’s surface becomes more viscous, allowing external particles, such as sand, to adhere to it. This phenomenon is facilitated by the plastic’s molten state, which acts like an adhesive. Figure 6d represents the final stage of this process, where the sand adheres to the plastic.

The degree of photodegradation directly affects the adhesion of external particles. P72, shown in Figure 6e, exhibited the highest adhesion, as evidenced by the incorporation of sand into the material after thermal treatment. On the other hand, as shown in Figure 6f, P130, which underwent more intense degradation, may have exhibited lower fusion due to the fragility of the polymer chains, reducing viscosity and, consequently, the adhesion of the sand.

The P130 sample, after thermal treatment, being more degraded, likely exhibits more broken polymer chains and lower structural cohesion. This may reduce its ability to form cohesive plastiglomerates and present sufficiently adhesive surfaces. Furthermore, structural fragility may result in a surface less prone to interacting with solid particles, such as sand.

These phenomena observed in microplastics, particularly those related to their interaction with particles in the environment, are highly relevant to studies on the environmental impact of microplastics. Modified microplastic particles, such as plastiglomerates, can alter their physical and chemical properties, which affects their dispersion and interaction with living organisms.^12,45^

The combined use of the backscattered Electron (BSE) mode Figure 7g and the secondary electron (SE) mode, Figure 7h, allows for a more detailed analysis of plastiglomerate formations. In the image obtained in BSE mode, there is greater contrast between the minerals and the sand, due to the higher atomic numbers of the elements present in these materials. The compositional contrast provided by BSE allows for the identification of regions of higher density (heavier minerals and sand particles), which appear brighter in the image. This behavior is explained by the greater reflection of electrons in materials with higher atomic numbers.^46^

**Figure 7:**
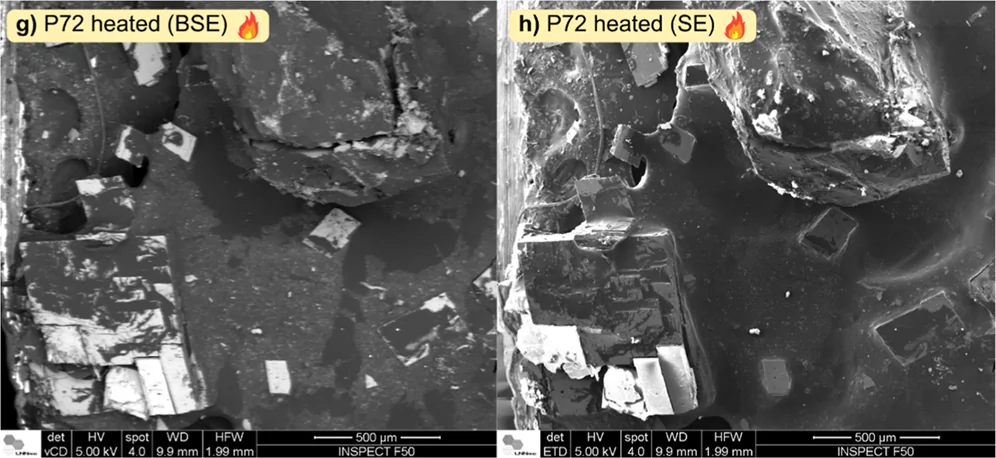
Scanned samples of heated MPs analyzed by SEM, where (g) shows MPs photodegraded for 72 h in backscattered electron (BSE) mode and (h) shows MPs photodegraded for 72 h in secondary electron (SE) mode, after thermal treatment.

On the other hand, in the image obtained in SE mode, the surface is analyzed based on secondary electron emission, which highlights the topography and the plastic coating. The presence of molten plastic, generally composed of carbon with a low atomic number, appears as darker areas in the image. This feature allows for a clearer identification of the extent of the plastic coating over the minerals and sand, confirming the formation of plastiglomerates.^47^

#### 3.3.2 Mass and Area

According to Table 3, all samples exhibited an increase in mass due to sand agglomeration after thermal treatment. Sample P72 showed the highest percentage of agglomeration, with an increase of 1659% compared to its initial weight. Next, sample P130 showed an increase of 912%, and finally, sample P0 showed an increase of 495%.

**Table 3:** Changes in Weight and Surface Area of Microplastic Samples before and after Thermal Treatment at 250 °C

parameters	P0	P72	P130
**prethermal treatment mass** **(g)**	0.0020 ± 0.0005	0.0029 ± 0.0003	0.0025 ± 0.0006
**post-thermal** **treatment mass** **(g)**	0.0099 ± 0.0025	0.0481 ± 0.0081	0.0228 ± 0.0037
**mass variation** **(Δ*w*)**	0.0079 ± 0.0021	0.0452 ± 0.0079	0.0203 ± 0.0030
**percentage increase** **(%)**	495%	1659%	912%
**prethermal treatment area** **(mm** ^**2**^ **)**	24.3 ± 0.0096	25.1 ± 0.0151	24.7 ± 0.0097
**post-thermal** **treatment area** **(mm** ^**2**^ **)**	6.9 ± 0.0145	18.8 ± 0.0571	24.1 ± 0.0040
**area variation** **(Δ*a*)**	17.4 ± 0.0049	6.3 ± 0.04200	0.6 ± 0.0057
**percentage decrease** **(%)**	72%	25%	2.4%

Based on Table 3 and Figure 8, a more pronounced increase in mass is observed in sample P72. This is because, in the early stages of photodegradation, such as at 72 h, the material still retains much of its structural integrity, exhibiting a more cohesive surface with characteristics that promote the adhesion of external particles. At this stage, sufficient chemical and physical changes occur to make the surface more reactive and irregular, but not to the point of causing significant disintegration. This balance between degradation and structural maintenance increases the material’s ability to incorporate and retain sand during heating.^48,49^

**Figure 8:**
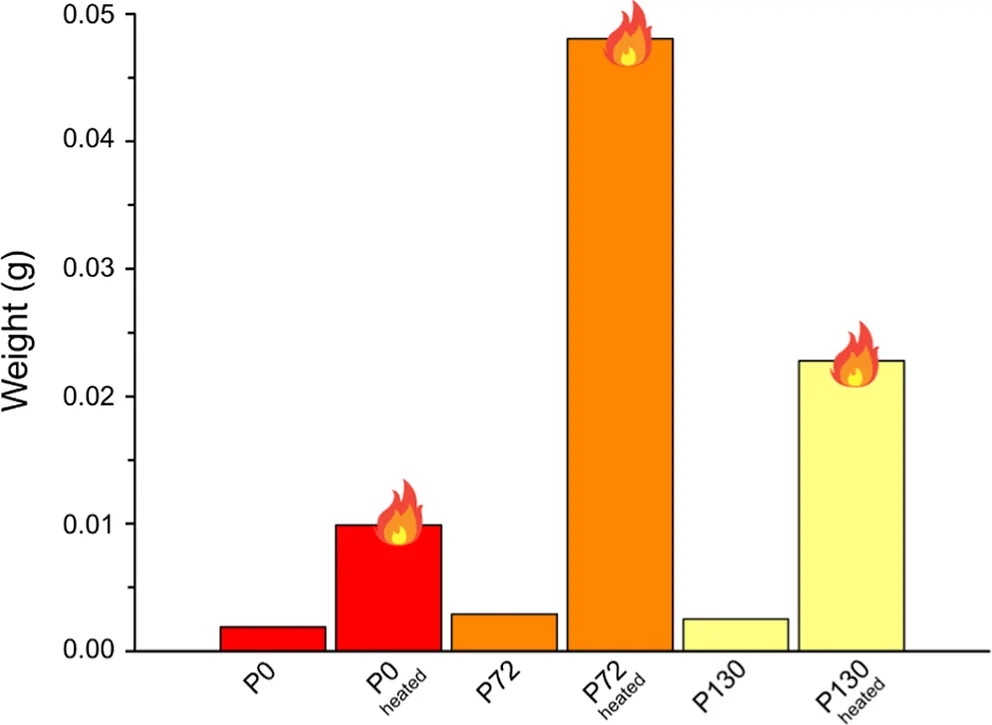
Mass of the MPs before and after thermal treatment.

According to Table 3 and Figure 9, all samples exhibited a reduction in size. Sample P0 showed the greatest percentage reduction, at 72%, followed by the sample photodegraded for 72 h, at 25%, and the 130 h sample, at 2.4% reduction, the latter showing the lowest percentage.

**Figure 9:**
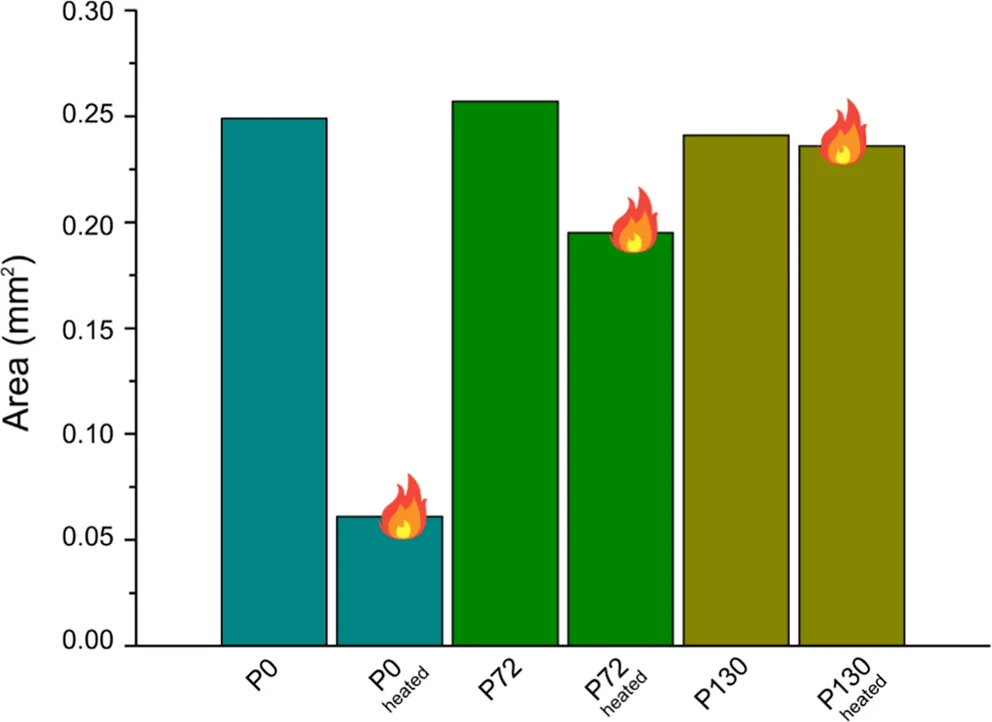
Area of the MPs sites before and after thermal treatment.

The difference in the variation of area reduction after thermal treatment among the samples can be explained by the structural changes caused by photodegradation. During photodegradation, the PE undergoes breaks in the polymer chain, forming functional groups such as carbonyl and hydroxyl groups. These changes make the material more brittle and susceptible to thermal stress.

Based on this, when analyzing the behavior of each sample, it is observed that P0 exhibits an intact polymeric structure, not yet modified by the effects of photodegradation. For this reason, the material remains more susceptible to thermal degradation when subjected to heating, resulting in a greater loss of surface area.^50,51^

In the P72 sample, the material already exhibits initial chemical changes, such as the formation of oxygen-containing groups (e.g., carbonyl groups), as well as physical changes on the surface. These modifications make the polymer stiffer and slightly more stable when heated than P0, reducing the rate of area loss. However, the photodegradation process is not yet as advanced as in the P130 sample.^52^

After 130 h of UV exposure, the material exhibits a higher concentration of photodegradation products and greater structural alteration along the polymer chains. These transformations make the polymer more thermally stable, resulting in a smaller reduction in surface area during thermal treatment, since the material has already undergone more intense oxidation and aging processes.^50,52^ A one-way ANOVA was performed, and it showed a highly significant effect of thermal treatment on mass variation (*F* = 78.95, *p* < 0.001). Subsequently, Tukey’s posthoc test was conducted, acordding Table 4, and the test indicated that all pairwise comparisons between the groups were statistically significant (*p* < 0.001), confirming that each thermal treatment condition resulted in distinct mass variations.

**Table 4:** Tukey’s HSD Post Hoc Test Comparing Samples Heated and Unheated^a^

comparison	mean difference	adjusted *p*-value	significance
P0 × P72	alta	<0.001	***
P0 × P130	moderada	<0.001	***
P72 × P130	alta	<0.001	***

a Significance levels are indicated as follows: ns, not significant (*p* ≥ 0.05); *p < 0.05; **p < 0.01; ***p < 0.001; ****p < 0.0001.

Figure 10 clearly illustrates the behavior of microplastics before and after thermal treatment, highlighting the trend of area reduction previously observed in the table. The box plot allows us to visualize the distribution of values and confirm the consistency of this decrease across all treatments. The graphical representation reinforces that P0 is more sensitive to heating, exhibiting greater variation and a greater loss of area. In contrast, the photodegraded samples exhibit more stable behavior, with less pronounced reductions. Thus, Figure 10 complements the quantitative data, providing a consolidated visualization of the trend already indicated in Table 1.

**Figure 10:**
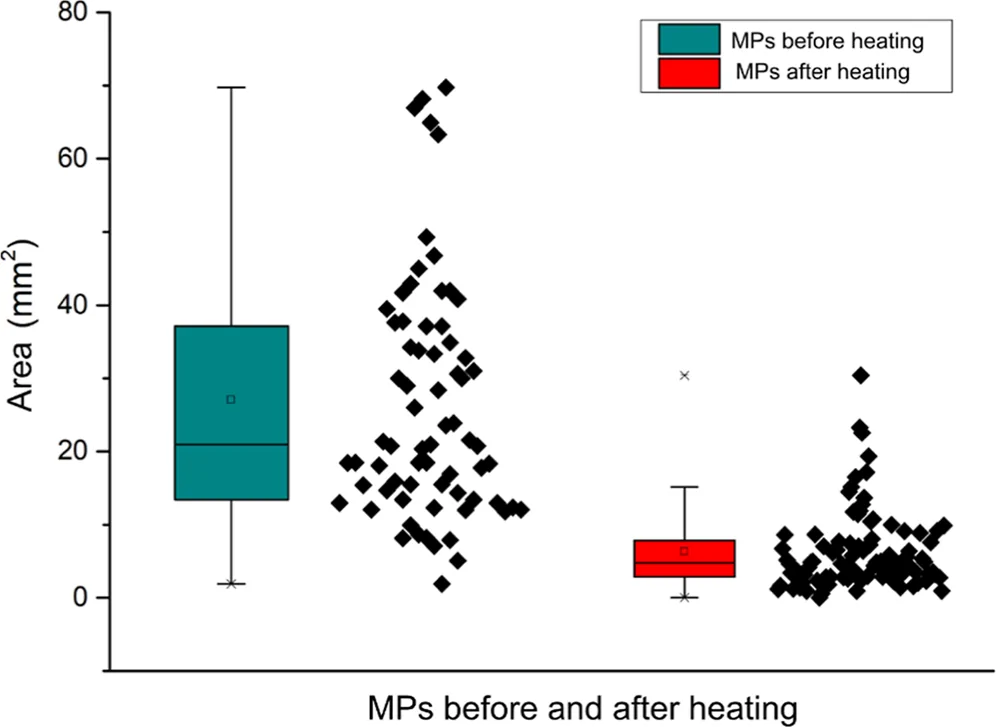
Box plot showing the behavior of the MPs before and after thermal treatment at 250 °C.

## 4 Conclusions

Thermal treatment at 250 °C promoted the formation of polyethylene–sand plastiglomerates in all samples. The highest sand adhesion was observed for the P72 sample, which exhibited a 1659% increase in mass, followed by P130, 912%, and P0, 495%. In contrast, the greatest reduction in surface area occurred for the pristine polyethylene (P0, 72%), whereas P72 and P130 exhibited reductions of 25% and 2.4%, respectively. Statistical analysis confirmed that both the degree of photodegradation and thermal treatment significantly affected the measured properties, with significant main effects and interaction between factors (Two-way ANOVA, *p* < 0.0001). Tukey’s post hoc test further demonstrated significant differences among all treatments (*p* < 0.001).

FTIR analyses revealed qualitative spectral changes consistent with polyethylene oxidation after photodegradation and thermal treatment, including the presence of carbonyl-, ester-, and hydroxyl-related bands. These observations are in agreement with the established literature and were used as complementary qualitative evidence of photodegradation within the scope of this study. Water contact angle measurements indicated significant changes in surface wettability after thermal treatment, although the variation observed for the P130 sample was comparatively small.

SEM observations qualitatively suggested the formation of surface cracks after photodegradation and confirmed the adhesion of sand particles to the polyethylene surface following thermal treatment, supporting the formation of plastiglomerates. Overall, the results demonstrate that the degree of prior photodegradation influences the interaction between polyethylene microplastics and mineral particles during heating, with moderately photodegraded polyethylene (P72) showing the greatest potential for plastiglomerate formation under the conditions evaluated.

## Supplementary Material



## Notes

Figures S1 and S2 showing the polyethylene MP experiment before and after heat treatment—FTIR spectrum of the carbonyl band for the samples before and after burning in Figure S3 (PDF)

## References

[1] Impactos ambientais e na saúde causados pelas queimadas no Brasil; ABC. https://www.abc.org.br/2024/10/01/impactos-ambientais-e-na-saude-causados-pelas-queimadas-no-brasil/ (accessed 2024–12–14).

[2] Brazil faces worst fires in 14 years – DW – 10/12/2024. dw.com. https://www.dw.com/en/brazil-faces-worst-fires-in-14-years/a-70475926 (accessed 2024–12–14).

[3] Wildfires continue to rise in Brazil’s main biomes in 2024. https://www.wwf.org.br/?90121/Wildfires-continue-to-rise-in-Brazils-main-biomes-in-2024 (accessed Dec 2024, 14).

[4] Silva P. S., Rodrigues J. A., Nogueira J., Moura L. C., Enout A., Cuiabália C., DaCamara C. C., Pereira A. A., Libonati R. (2024). Joining Forces to Fight
Wildfires: Science and Management in a Protected Area of Pantanal,
Brazil. Environ. Sci. Policy.

[5] Staff, C. B. Climate change made the ‘supercharged’ 2024 Pantanal wildfires 40% more intense; Carbon Brief. https://www.carbonbrief.org/climate-change-made-the-supercharged-2024-pantanal-wildfires-40-more-intense/ (accessed 2024–10–15).

[6] Bustamante M. M. d. C. (2025). Climate
Change and Children’s Health: Resilience Challenges for Brazil. J. Pediatr..

[7] Rizzo L. V., Rizzo M. C. F. V. (2025). Wildfire Smoke
and Health Impacts: A Narrative Review. J. Pediatr.
(Rio J.).

[8] Corcoran P. L., Moore C. J., Jazvac K. (2014). An Anthropogenic
Marker Horizon in the Future Rock Record. GSA
Today.

[9] Santos F. A., Diório G. R., Guedes C. C. F., Fernandino G., Giannini P. C. F., Angulo R. J., de Souza M. C., César-Oliveira M. A. F., dos
Santos Oliveira A. R. (2022). Plastic Debris Forms: Rock Analogues Emerging from
Marine Pollution. Mar. Pollut. Bull..

[10] Goswami P., Bhadury P. (2023). First Record of an
Anthropocene Marker Plastiglomerate in Andaman Island, India. Mar. Pollut. Bull..

[11] Rakib M. R. J., De-la-Torre G. E., Jolly Y. N., Al Nahian S., Khan N. I., Idris A. M. (2023). First Record
of Plastiglomerate and Pyroplastic Pollution in the World’s
Longest Natural Beach. Sci. Total Environ..

[12] Utami D. A., Reuning L., Schwark L., Friedrichs G., Dittmer L., Nurhidayati A. U., Al Fauzan A., Cahyarini S. Y. (2023). Plastiglomerates from Uncontrolled
Thermal treatment of Plastic Waste on Indonesian Beaches Contain High
Contents of Organic Pollutants. Sci. Rep..

[13] Sebastião G. I.
A., Rani-Borges B., Dipold J., Freitas A. Z., Wetter N. U., Ando R. A., Waldman W. R. (2025). Forensic Determination of Adhesive Vinyl Microplastics
in Urban Soils. J. Environ. Manage..

[14] Boeing G. A. N. S., Provase M., Tsukada E., Salla R. F., Waldman W. R., Abdalla F. C. (2024). Spray Paint-Derived Microplastics
and Incorporated Substances as Ecotoxicological Contaminants in the
Neotropical Bumblebee Bombus Atratus. Environ.
Toxicol. Pharmacol..

[15] do Amparo S. Z. S., Carvalho L. de O., Silva G. G., Viana M. M. (2023). Microplastics as Contaminants in the Brazilian Environment:
An Updated Review. Environ. Monit. Assess..

[16] Kedzierski M., Cirederf-Boulant D., Palazot M., Yvin M., Bruzaud S. (2023). Continents of Plastics:
An Estimate of the Stock of Microplastics in Agricultural Soils. Sci. Total Environ..

[17] Astner A. F., Gillmore A. B., Yu Y., Flury M., DeBruyn J. M., Schaeffer S. M., Hayes D. G. (2023). Formation, Behavior, Properties and Impact of Micro-
and Nanoplastics on Agricultural Soil Ecosystems (A Review). NanoImpact.

[18] Ihenetu S. C., Li G., Mo Y., Jacques K. J. (2024). Impacts of Microplastics and Urbanization on Soil Health:
An Urgent Concern for Sustainable Development. Green Anal. Chem..

[19] Khalid N., Aqeel M., Noman A., Fatima Rizvi Z. (2023). Impact of Plastic Mulching as a Major Source of Microplastics
in Agroecosystems. J. Hazard. Mater..

[20] Grifoni M., Pellegrino E., Arrighetti L., Bronco S., Pezzarossa B., Ercoli L. (2024). Interactive Impacts
of Microplastics and Arsenic on Agricultural Soil and Plant Traits. Sci. Total Environ..

[21] Wang C., Luo Q., Zhang J., Zhang X., Yang N., Feng L. (2023). Toxic Effects of Microplastics
and Nanoplastics on Plants: A Global Meta-Analysis. Environ. Pollut..

[22] Xu M., Ma W., Yao Y., Xu Q., Du W., Yin Y., Ji R., Wang X., Guo H. (2024). Investigation of the Effects of Polyethylene Microplastics at Environmentally
Relevant Concentrations on the Plant-Soil-Microbiota System: A Two-Year
Field Trial. Sci. Total Environ..

[23] Frias J. P. G. L., Nash R. (2019). Microplastics: Finding
a Consensus on the Definition. Mar. Pollut.
Bull..

[24] Cacuro T. A., Freitas A. S. M., Waldman W. R. (2018). Demonstration
of Polymer Photodegradation Using a Simple Apparatus. J. Chem. Educ..

[25] Bradstock R. A., Auld T. D. (1995). Soil Temperatures During Experimental
Bushfires in Relation to Fire Intensity: Consequences for Legume Germination
and Fire Management in South-Eastern Australia. J. Appl. Ecol..

[26] Miotliński K., Tshering K., Boyce M. C., Blake D., Horwitz P. (2023). Simulated
Temperatures of Forest Fires Affect Water Solubility in Soil and Litter. Ecol. Indic..

[27] Rascio I. (2022). Evidence of hexavalent
chromium formation and changes of Cr speciation after laboratory-simulated
fires of composted tannery sludges long-term amended agricultural
soils. J. Hazard. Mater..

[28] Zavala L. M., Granged A. J. P., Jordán A., Bárcenas-Moreno G. (2010). Effect of
Thermal treatment Temperature on Water Repellency and Aggregate Stability
in Forest Soils under Laboratory Conditions. Geoderma.

[29] Han, J. ; Kamber, M. ; Pei, J. Data Preprocessing. In Data Mining, 3 ed.; Han, J. , Kamber, M. , Pei, J. , Eds.The Morgan Kaufmann Series in Data Management Systems; Morgan Kaufmann: Boston, 2012; pp 83–124.

[30] Gomes R., Fernandes A. N., Waldman W. R. (2024). How to Measure Polymer Degradation?
An Analysis of Authors’ Choices When Calculating the Carbonyl
Index. Environ. Sci. Technol..

[31] Chai C., Liang H., Yao R., Wang F., Song N., Wu J., Li Y. (2023). Photocatalytic
Degradation of Polyethylene and Polystyrene Microplastics by α-Fe2O3/g-C3N4. Environ. Sci. Pollut. Res..

[32] Chowdhury T., Wang Q., Enyoh C. E. (2022). Degradation
of Polyethylene Terephthalate Microplastics by Mineral Acids: Experimental,
Molecular Modelling and Optimization Studies. J. Polym. Environ..

[33] Lozano Y. M., Gordillo-Rocha H., Waldman W. R., Rillig M. C. (2024). Photodegradation
Modifies Microplastic Effects on Soil Properties and Plant Performance. J. Appl. Ecol..

[34] Piao M., Teng H., Zhao L., Du H. (2024). Recent Catalytic Technologies
for Microplastics Removal in Water: Current Status. Water, Air, Soil Pollut..

[35] Fa W., Wang J., Ge S., Chao C. (2020). Performance of Photo-Degradation
and Thermo-Degradation of Polyethylene with Photo-Catalysts and Thermo-Oxidant
Additives. J. Polym. Sci. (N. Y. NY U. S.).

[36] Rodríguez Chialanza M., Favre Samarra S., Pérez Parada A. (2022). Modeling Microplastic with Polyethylene (PE) Spherical
Particles: A Differential Scanning Calorimetry Approach for Quantification. Environ. Sci. Pollut. Res..

[37] Chia, W. ; Ng, Y. H. ; Ong, C. Y. FTIR and SEM Study on the Degradation of Microplastics. In IRC-SET 2020; Guo, H. , Ren, H. , Kim, N. , Eds.; Springer: Singapore, 2021; pp 539–546.

[38] Xie A., Jin M., Zhu J., Zhou Q., Fu L., Wu W. (2023). Photocatalytic Technologies
for Transformation and Degradation of Microplastics in the Environment:
Current Achievements and Future Prospects. Catalysts.

[39] Zvekic M., Richards L., Tong C., Krogh E. T. (2022). Characterizing
Photochemical Ageing Processes of Microplastic Materials Using Multivariate
Analysis of Infrared Spectra. Environ. Sci.
Process. Impacts.

[40] Campanale C., Savino I., Massarelli C., Uricchio V. F. (2023). Fourier Transform Infrared Spectroscopy
to Assess the Degree of Alteration of Artificially Aged and Environmentally
Weathered Microplastics. Polymers.

[41] Celina M. C. (2013). Review
of Polymer Oxidation and Its Relationship with Materials Performance
and Lifetime Prediction. Polym. Degrad. Stab..

[42] Hoseini M., Stead J., Bond T. (2023). Ranking the
Accelerated Weathering of Plastic Polymers. Environ. Sci. Process. Impacts.

[43] Kumari S., Yadav D., Yadav S., Selvaraj M., Sharma G., Karnwal A., Yadav S. (2025). From Macro to Micro: The Key Parameters
Influencing the Degradation Mechanism and the Toxicity of Microplastics
in the Environment. Polym. Degrad. Stab..

[44] Ojha, M. D. ; Skariyachan, S. Degradation of Low-Density Polyethylene by Physical, Chemical, and Microbial-Based Approaches. In Sustainable Microbial Technology for Synthetic and Cellulosic Microfiber Bioremediation; Das, A. P. , Behera, I. D. , Bhanja, D. , Eds.; Springer Nature Switzerland: Cham, 2024; pp 259–274.

[45] Plastic Rocks Discovered on India’s East Coast. Nat. India 2024. DOI: 10.1038/d44151-024-00102-x.

[46] Lv S., Li Y., Yuan Q., Lu Y., Ye Y., Zhong Y., Liu R., Zhao S., Xia J., Zeng L., Shao Z. (2024). Influence of Microplastics on the
Structure and Function of Deep-Sea Communities during Long-Term Enrichment
Processes. Front. Mar. Sci..

[47] Narwal N., Katyal D. (2025). The Abundance and Analytical Characterization of Microplastics
in the Surface Water of Haryana, India. Microsc.
Res. Technol.

[48] Zakaria, S. F. ; Rosnan, S. M. Photodegradation of Materials: An Overview. In Proceedings of the International Symposium on Research of Arts, Design and Humanities (ISRADH 2014); Hassan, O. H. , Abidin, S. Z. , Anwar, R. , Kamaruzaman, M. F. , Eds.; Springer: Singapore, 2015; pp 171–178.

[49] Zhang W., Shen J., Guo X., Wang K., Jia J., Zhao J., Zhang J. (2024). Comprehensive Investigation into
the Impact of Degradation of Recycled Polyethylene and Recycled Polypropylene
on the Thermo-Mechanical Characteristics and Thermal Stability of
Blends. Molecules.

[50] Alavian Petroody S. S., Hashemi S. H., Škrlep L., Mušič B., van Gestel C. A. M., Sever Škapin A. (2023). UV Light Causes
Structural Changes in Microplastics Exposed in Bio-Solids. Polymers.

[51] Bandaru S., Ravipati M., Busi K. B., Phukan P., Bag S., Chandu B., Dalapati G. K., Biring S., Chakrabortty S. (2024). A Review on the Fate of Microplastics:
Their Degradation and Advanced Analytical Characterization. J. Polym. Environ..

[52] Lv M., Jiang B., Xing Y., Ya H., Zhang T., Wang X. (2022). Recent Advances in the Breakdown of Microplastics: Strategies and
Future Prospectives. Environ. Sci. Pollut. Res..

