# Supplementary material for: Thermal Effect on the Formation of Polyethylene Greenhouse Plastiglomerates from Microplastics
Source: ACS Omega. 2026 Aug 3;11(32):48542–51. doi: 10.1021/acsomega.6c05760 (PMC13491503; doi:10.1021/acsomega.6c05760)
Supplement: Supplementary file 1 [file ao6c05760_si_001.pdf]

Thermal effect on the formation of polyethylene greenhouse plastiglomerates from microplastics

Luísa Carvalho Pereira Araújo[1*]
Fábio Minoru Yamaji[1]
Franciane Pádua[1]

[1]Federal University of São Carlos (UFSCar), Campus of Sorocaba
Department of Environmental Sciences, Rodovia João Leme dos Santos, km 110, Sorocaba, SP, Brazil, 18052-780

Supporting Information for Publication

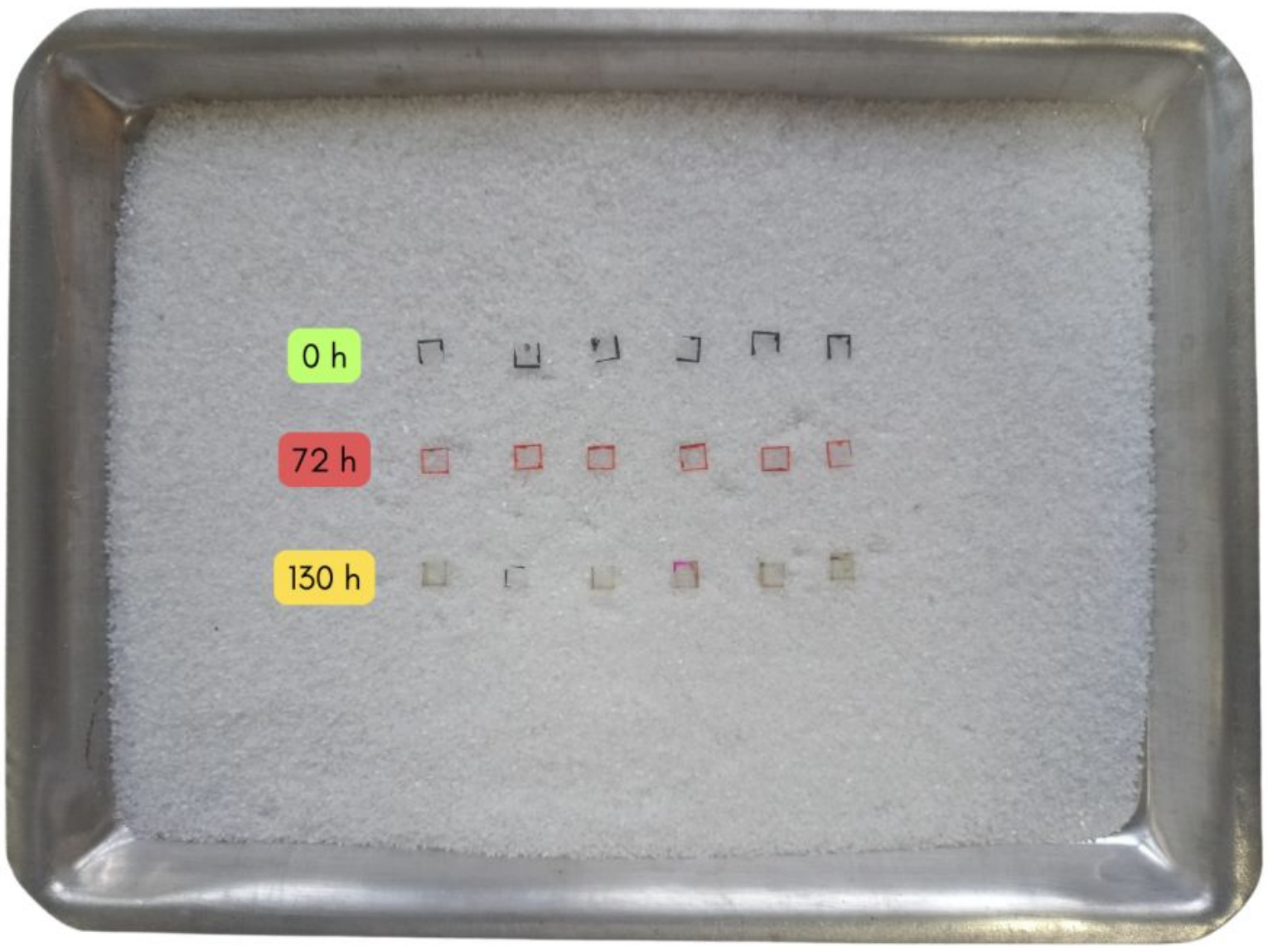

Figure S1 – Samples of polyethylene microplastics from an oven before thermal treatment at 250 ºC, degraded in 0 h, 72 h and 130 h, respectively, as shown in the image.

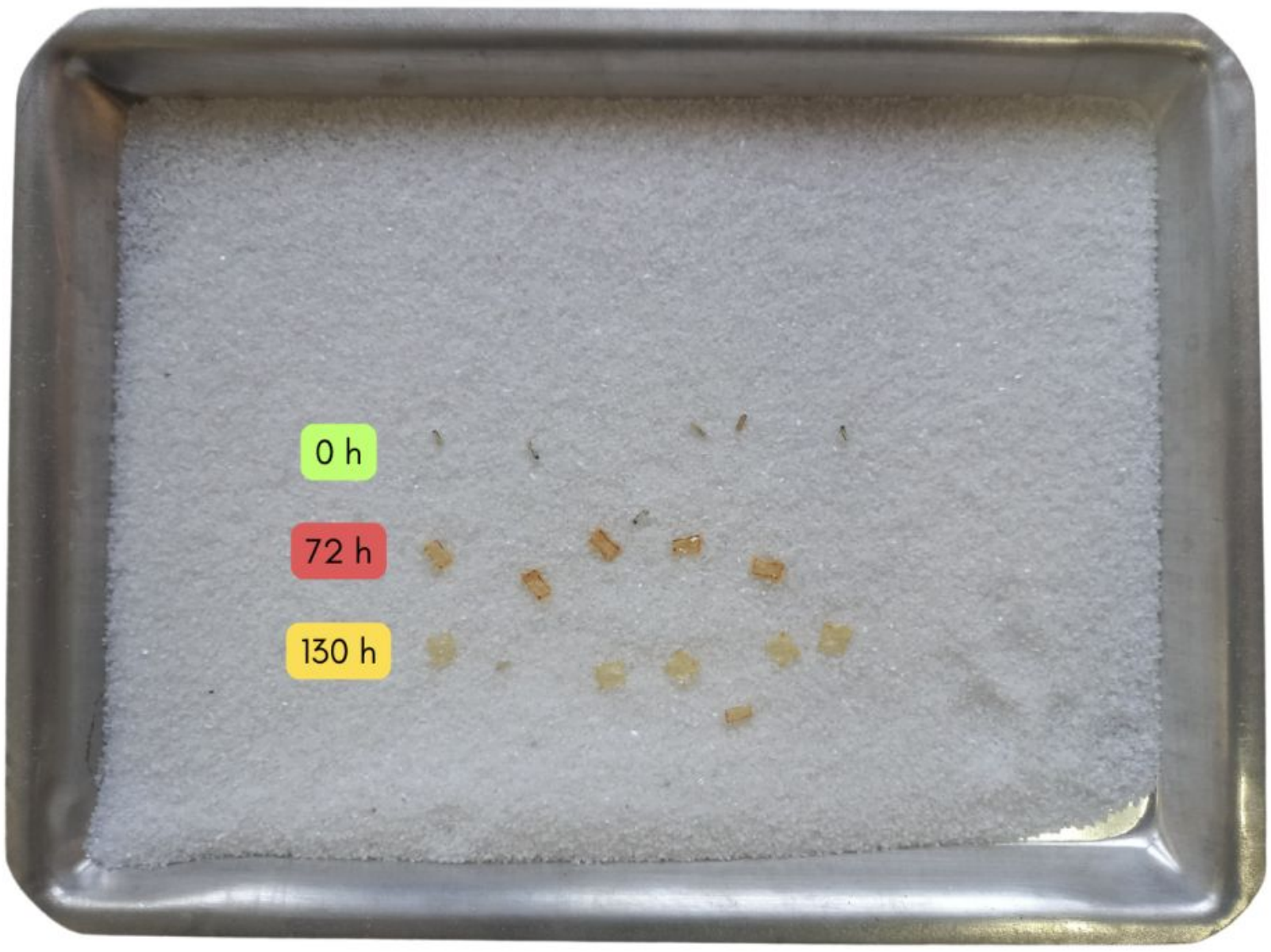

Figure S2 - Samples of polyethylene microplastics from an oven after thermal treatment at 250 ºC.

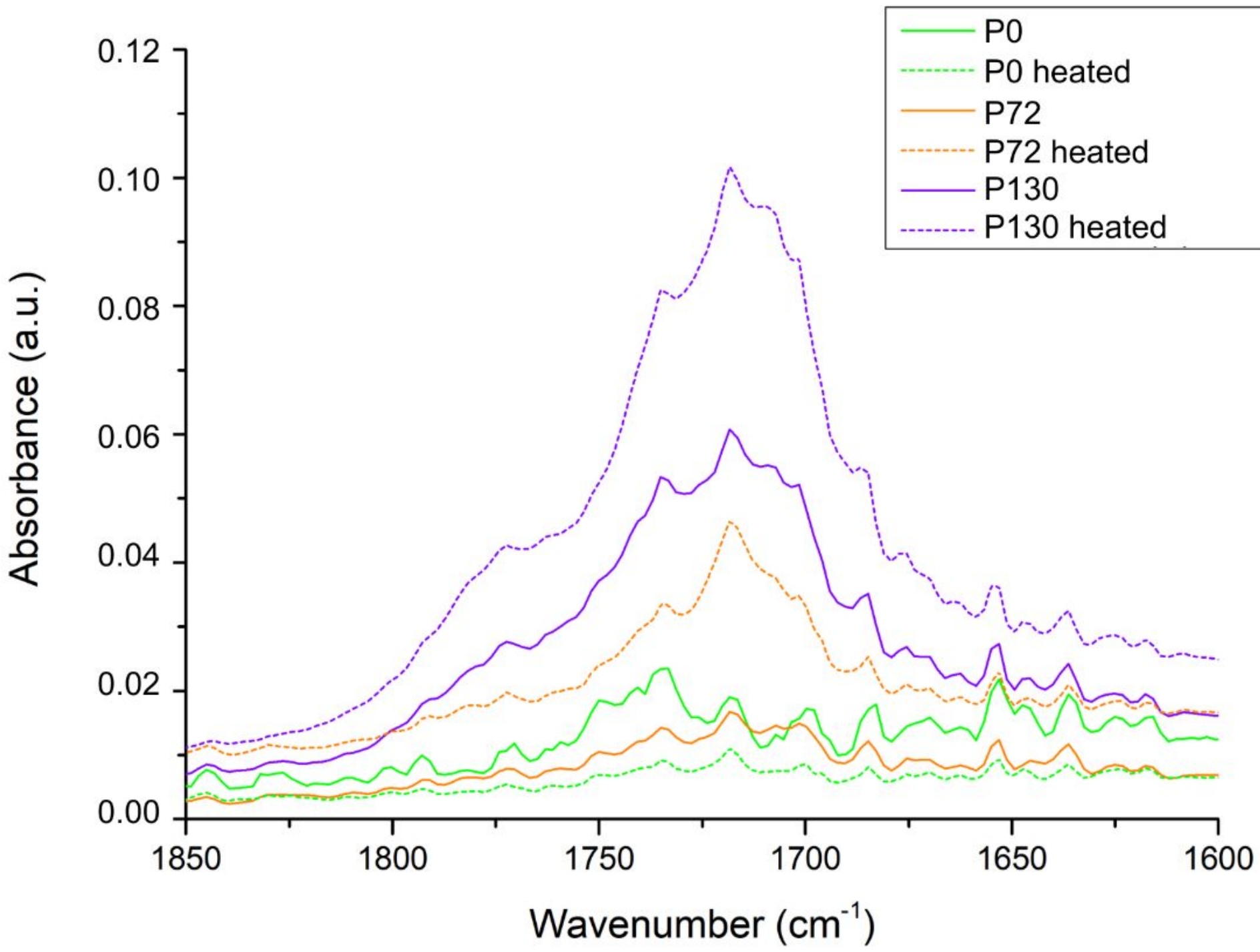

Figure S3 – Spectrum of carbonyl groups before and after thermal treatment at 250 ºC.
